# The effect of a pre- and post-operative exercise programme versus standard care on physical fitness of patients with oesophageal and gastric cancer undergoing neoadjuvant treatment prior to surgery (The PERIOP-OG Trial): Study protocol for a randomised controlled trial

**DOI:** 10.1186/s13063-020-04311-4

**Published:** 2020-07-13

**Authors:** Roisin Tully, Lisa Loughney, Jarlath Bolger, Jan Sorensen, Oliver McAnena, Chris G. Collins, Paul A. Carroll, Mayilone Arumugasamy, Tomas J. Murphy, William B. Robb, on behalf of the PERIOP OG Working Group, Wendy Hickey, Claire Coleman, Louise Buckley, Eileen Lombard, Noel McCaffrey, Pamela Gallagher, Claire Timon, Patricia Kearney, Aoife Quinn, Emma Houlihan, D. J. O’Dwyer, Catherine Woods, Raymond O’Connor, Sinead Kelly, Brona Kehoe, Mark McManus, Austin Twomey

**Affiliations:** 1 Department of Upper GI Surgery, Beaumont Hospital, Dublin, Ireland; 2 The Royal College of Surgeons in Ireland, St. Stephens Green, Dublin, Ireland; 3 ExWell Medical, Santry Sports Link, Dublin, Ireland; 4 Department of Upper GI Surgery, University Hospital, Galway, Ireland; 5 Department of Upper GI Surgery, Mercy University Hospital, Cork, Ireland

**Keywords:** Neoadjuvant chemoradiotherapy, Exercise training, Physical fitness, Quality of life, Cancer, Oesophagogastric

## Abstract

**Background:**

Advances in peri-operative oncological treatment, surgery and peri-operative care have improved survival for patients with oesophagogastric cancers. Neoadjuvant cancer treatment (NCT) reduces physical fitness, which may reduce both compliance and tolerance of NCT as well as compromising post-operative outcomes. This is particularly detrimental in a patient group where malnutrition is common and surgery is demanding. The aim of this trial is to assess the effect on physical fitness and clinical outcomes of a comprehensive exercise training programme in patients undergoing NCT and surgical resection for oesophagogastric malignancies.

**Methods:**

The PERIOP-OG trial is a pragmatic, multi-centre, randomised controlled trial comparing a peri-operative exercise programme with standard care in patients with oesophagogastric cancers treated with NCT and surgery. The intervention group undergo a formal exercise training programme and the usual care group receive standard clinical care (no formal exercise advice). The training programme is initiated at cancer diagnosis, continued during NCT, between NCT and surgery, and resumes after surgery. All participants undergo assessments at baseline, post-NCT, pre-surgery and at 4 and 10 weeks after surgery. The primary endpoint is cardiorespiratory fitness measured by demonstration of a 15% difference in the 6-min walk test assessed at the pre-surgery timepoint. Secondary endpoints include measures of physical health (upper and lower body strength tests), body mass index, frailty, activity behaviour, psychological and health-related quality of life outcomes. Exploratory endpoints include a health economics analysis, assessment of clinical health by post-operative morbidity scores, hospital length of stay, nutritional status, immune and inflammatory markers, and response to NCT. Rates of NCT toxicity, tolerance and compliance will also be assessed.

**Discussion:**

The PERIOP-OG trial will determine whether, when compared to usual care, exercise training initiated at diagnosis and continued during NCT, between NCT and surgery and then during recovery, can maintain or improve cardiorespiratory fitness and other physical, psychological and clinical health outcomes. This trial will inform both the prescription of exercise regimes as well as the design of a larger prehabilitation and rehabilitation trial to investigate whether exercise in combination with nutritional and psychological interventions elicit greater benefits.

**Trial registration:**

ClinicalTrials.gov: NCT03807518. Registered on 1 January 2019

## Background

Recent advances in peri-operative oncological treatments have led to survival benefits for patients with locally advanced oesophagogastric cancers [1–3]. In spite of the benefits, neoadjuvant cancer therapy (NCT), due to its inherent toxicity, can significantly impact on patients’ fitness for subsequent surgical resection [4–6]. Reduced physical fitness is associated with poor tolerance of peri-operative oncological treatment, increased toxicity and compromised peri-operative outcomes [4–6]. Evidence exists that reduced physical fitness in the pre-operative period is also a negative predictor of long-term survival in oesophagogastric cancer [7].

Peri-operative prehabilitation and rehabilitation have been shown to be effective in cardiothoracics, orthopaedics and abdominal cancers [8–10]. The widespread use of neoadjuvant therapy in oesophagogastric cancers offers a distinct window during which prehabilitation can be undertaken. This cancer group does however present unique challenges, as patients tend to be older, have pre-existing co-morbidity and often present with nutritional compromise. Importantly, any exercise prescription should not negatively impact upon the physiological reserve of patients undergoing NCT followed by resectional oesophagogastric surgery.

Despite improvements in surgical techniques for oesophagogastric cancer, peri-operative morbidity remains significant [11–15]. Post-operative complications result in increased utilisation of critical care, prolonged hospital stay and long-term adverse events [13, 16]. Peri-operative morbidity is now also increasingly recognised to be associated with reduced overall and cancer-specific survival [17]. Patients who are less physically fit at the time of operation have a higher incidence of post-operative morbidity and mortality [18] and hence any strategy which can reduce physical decline or improve physical conditioning between cancer diagnosis and surgery is worthy of investigation.

Following oesophagogastric cancer surgery rehabilitation efforts appear to improve cardiorespiratory fitness and quality of life without compromising nutritional status [19]. How pre-operative programmes impact on patient outcomes is less defined. Evidence that inspiratory muscle, aerobic and resistance training may reduce peri-operative morbidity is limited [20], and both prehabilitation and rehabilitation may improve functional outcomes [20]. The optimal peri-operative exercise strategy remains ill-defined, and what form exercise interventions should take is unclear. Whether programmes should be supervised, home-based or a combination of both is also unclear, and measures of compliance are not established [20]. Whilst some evidence exists that health-related quality of life (HRQoL) may be improved by post-operative exercise programmes [19], data on HRQoL measures from pre-operative interventions have yet to demonstrate significant improvements. A number of small trials and cohort studies are underway which may help to bridge the gap in knowledge [21–27].

The primary aim of the PERIOP-OG trial is to investigate the effect of a community-based exercise training programme, delivered throughout NCT and prior to surgery compared to usual care. The secondary aim is to investigate the effect of a 6 week post-operative exercise programme in the same cohort. The trial will comprehensively study the effects of exercise on physical, psychological and clinical health outcomes in patients with locally advanced oesophagogastric cancer undergoing neoadjuvant treatment followed by curative surgery.

## Methods/Design

The PERIOP-OG trial is a prospective and pragmatic randomised controlled multi-centre superiority trial that compares a programme of peri-operative exercise with standard care in patients with oesophagogastric cancer undergoing NCT followed by surgery. Three university teaching hospitals in Ireland (Beaumont Hospital, Dublin; The Mercy University Hospital, Cork; and Galway University Hospital, Galway) are recruiting to the trial with the exercise training programme delivered in seven exercise centres nationwide. The exercise training is delivered through ExWell Medical, a chronic illness exercise and rehabilitation service, and its exercise partners nationwide.

Lead exercise personnel perform assessments after receiving standardised training by lead study coordinators (LL and RT).

**Fig. 1:**
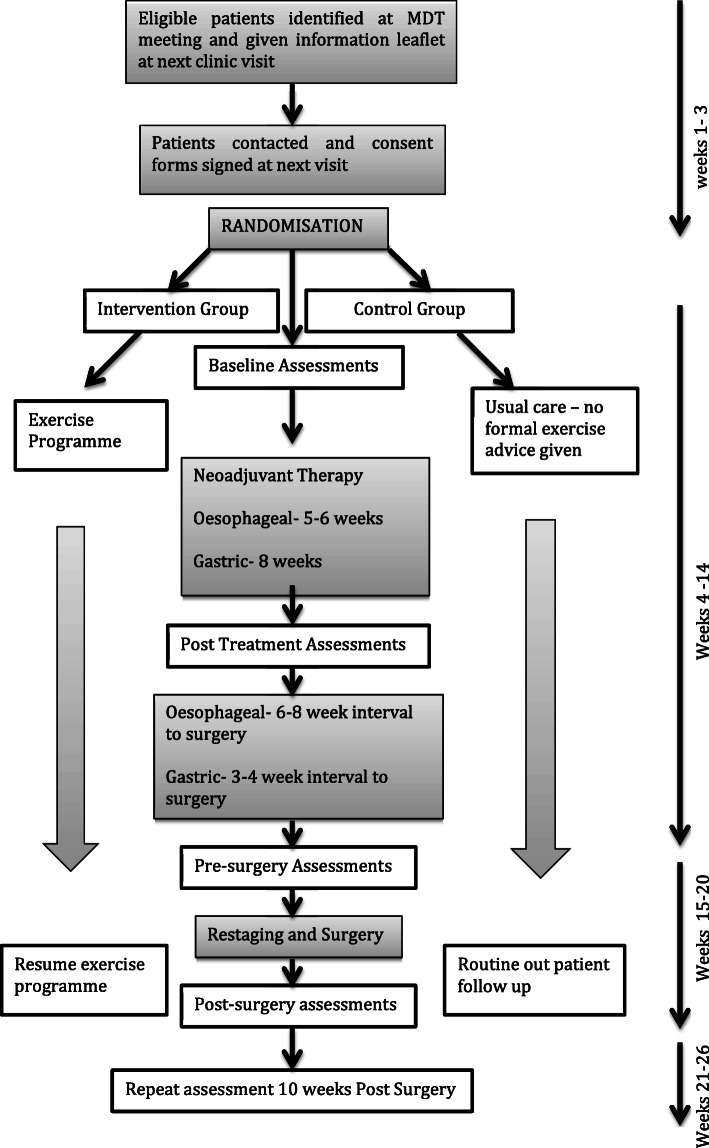
The PERIOP-OG trial algorithm

An algorithm of the clinical pathway and the timepoints for assessments are shown in Fig. 1. Ethical approval for this study has been received in each participating site prior to study commencement, and the trial is registered with clinicaltrials.gov (NCT: NCT0380751).

### Study objectives

The primary objective of the PERIOP-OG trial is to demonstrate that a structured community-based exercise programme will result in a clinically significant increase in cardiorespiratory fitness pre-surgery when compared to a standard care control group. Cardiorespiratory fitness is assessed using a 6-min walk test (6MWT) at five timepoints in the trial:baseline, post-NCT, pre-surgery, and 4 weeks and 10 weeks after surgery.

Secondary aims include assessing whether exercise training improves other physical health outcomes: upper and lower body strength tests, activity behaviour monitoring, body mass index and frailty. Psychological health is assessed using a series of questionnaires: EQ-5D-5 L Health Questionnaire, Functional Assessment of Cancer Therapy (FACT-E), General Self Efficacy (GSE), Pearlin Mastery Scale (PMS), Surgical Fear Questionnaire (SFQ) and general optimism using the Life Orientation Test-Revised (LOT-R) tool as well as semi-structured interviews.

Exploratory end-points include assessment of post-operative morbidity (Clavien-Dindo classification and as agreed upon by the Esophagectomy Complications Consensus Group (ECCG) [28], the Comprehensive Complication Index, hospital length of stay, nutritional status (serum albumin, sarcopenia score and Foodbook-24), inflammatory markers, cancer staging and response to NCT and a medico-economics analysis of cost effectiveness of the exercise intervention on reducing health care costs and burden. Additionally, rates of NCT toxicity, tolerance and compliance will be measured.

### Participants

Eligibility criteria include the following: age ≥ 18 years, multidisciplinary team (MDT) referral for neoadjuvant chemotherapy or neoadjuvant chemoradiotherapy prior to oesophagectomy or gastrectomy; confirmed adenocarcinoma or squamous cell cancer of the oesophagus, oesophago-gastric junction or stomach; and if oesophageal cancer, then the tumours must be more than 5 cm below the crico-pharyngeus muscle.

Exclusion criteria include the following: inability to give informed consent, inability to participate in the exercise training (unable to perform 6MWT), patients undergoing primary surgery, distant metastatic disease, or previous or concomitant malignancy that would interfere with this treatment protocol and pregnancy.

### Recruitment and randomisation

The PERIOP-OG trial is currently recruiting (start date 1 March 2019, proposed end date July 2020). All potentially eligible patients are identified in each centre’s MDT and are approached for inclusion at diagnosis before NCT has started. Eligible patients are given an information leaflet and then are contacted 72 h later to confirm participation. A baseline assessment visit is then scheduled whereby informed consent is taken, and the randomisation group revealed. Participants are randomised using central data management to generate a random allocation sequence (1:1). Due to the nature of the study, blinding of patients, data collectors and physiological assessors is not possible but the treating surgeons and their teams are blinded to randomisation, as is the primary analyst.

### Nutrition

Malnutrition is common in patients diagnosed with oesophagogastric cancers. All participants enrolled in the PERIOP-OG trial follow a standardised nutritional pathway of care. All three participating centres have specialist dieticians who are highly trained and dedicated to the care of oesophagogastric cancer patients. All patients have a dietician assessment at the time of diagnosis and an individualised dietary plan with appropriate supplementation structured to ensure sufficient calorie and protein supplementation. Peri-operative feeding adjuncts (percutaneous enteral feeding or total parenteral nutrition) will be recorded on an individual basis.

### Usual care control group

The usual care control group (no formal exercise training) receive routine care throughout their cancer pathway. No specific advice about exercise training is offered. Activity monitors are worn for a period of 7 days to measure activity behaviours in both groups at each timepoint.

### Exercise intervention group

The exercise-training programme starts before NCT (if time allows), continues throughout NCT, and following completion of NCT up to the point of surgery and resumes for 6 weeks after surgery once patients are deemed clinically fit. The exercise-training programme is based on experience gained from a previous feasibility study performed by our own team.

Participants in the exercise group are offered an option to participate in either a centre-based exercise programme (CBEP) or a home-based exercise programme (HBEP). This is to cater for all patients as the time-dense schedules of NCT regimens and the long distances some patients have to travel to their treating hospitals often pose a challenge in exercise prescribing. All participants in the exercise group are provided with an exercise programme pack which includes a manual exercise handbook, a Fitbit, a rate of percieved exertion (RPE) scale and a physical activity diary. They are also given a link to an online motivational video developed specifically for the PERIOP-OG trial.

The HBEP is offered for patients where access to an exercise centre is difficult due to remote or rural living. An individualised exercise prescription is provided initially at the baseline assessment and is reviewed at subsequent assessments. The HBEP involves undertaking exercise independently, however following the baseline assessment participants are educated in aerobic and resistance exercises, and they complete a 10-min exercise session on the cycle ergometer under the supervision of their personal trainer. This provides an understanding of what exercise intensity level they should aim to achieve during aerobic exercise at home, which is guided by the use of the RPE scale. Additionally, participants are instructed on resistance exercises (i.e., weight selection, technique, breathing and rest periods). HBEP participants receive a weekly telephone call, using a structured proforma, to assess adherence to the programme and to amend the programme if necessary. Participants feedback their daily step count and physical activities undertaken each week. All conversations and the duration of each phone call are documented in the participant case report forms. HBEP compliance is self-reported by the participant using a log diary which is returned to the lead researcher at the end of the trial.

The CBEP takes place in seven exercises centres nationwide. Compliance with the CBEP is recorded by number of sessions attended.

### Exercise training protocol

The delivery of the CBEP and HBEP is described using the FITT principle (frequency, intensity, time and type of exercise training) [29].

#### Frequency

Participants are asked to undertake two to three structured exercise training sessions per week during NCT and three exercise training sessions per week thereafter.

#### Intensity

Exercise sessions may include interval or continuous training (based on individual ability). Interval training involves a series of exercises repeated at moderate and high intensities and continuous training involves moderate intensity exercise for the entire duration of the exercise period.

Interval training of moderate and high intensities is prescribed using the RPE scale (13: somewhat hard to 15: hard) and continuous exercise training programme is prescribed using the RPE scale (13: somewhat hard).

#### Time

The first interval (moderate to high intensity) exercise session is 30 min: 5-min warm-up followed by four repeated bouts of moderate intensity (3 min) to high intensity (2 min) intervals and 5-min cool down. The first continuous exercise session is also of 30 min duration: 5 min warm-up, 20 min of continuous moderate intensity exercise and 5 min cool down.

The second and subsequent sessions are 40 min long and include a 5-min warm-up, followed by six bouts of moderate intensity (3 min) to high intensity (2 min) intervals and a 5-min cool down. The second continuous exercise session is made up of a 5-min warm-up, 30 min of continuous moderate intensity and a 5-min cool down. Post-operatively, participants resume exercising initially for 20-min sessions and increase the duration of exercise by 10 min per week until the pre-operative timings are achieved.

#### Type

CBEP or HBEP participants with access to gym equipment may include the use of any of the following equipment: upright cycle ergometer, recumbent cycle ergometer, treadmill, elliptical ergometer, and rowing ergometer, depending on patient preference. HBEP participants without gym access may use a combination of walking, jogging or cycling.

Resistance training involves a circuit of six to ten stations for alternating upper and lower body exercises as outlined in the home-based exercise manual handbook.

#### Progression

During NCT, no progression occurs in the exercise intensity. In the time window between completing NCT and surgery, exercise intensity is progressed every five sessions (if the participant is tolerating the exercise sessions well). Post-operatively, exercise is progressed by time (as previously outlined) and also by intensity at 8 weeks following surgery.

### Outcome measurements

Table 1 shows the timepoints of assessments of the outcome measures as part of the PERIOP-OG trial: baseline, post NCT, pre-surgery, 4 weeks post-surgery and 6 weeks later.

**Table 1:** Timeline of assessments in the PERIOP-OG trial

Outcomes	Assessment measure	Baseline	Post NCT	Pre-op	Day 3 post-op	Day 5 post-op	4 weeks post-op	10 weeks post -op
**Primary Endpoint**- Cardiorespiratory fitness	6 MWT	X	X	X			X	X
**Secondary Endpoints**								
Physical Health-Strength	Sit to stand test Grip strength	X	X	X			X	X
Activity Behaviours	Accelerometer	X	X	X			X	X
Body Composition	BMI	X	X	X			X	X
Psychological Health-Optimism	LOT-R	X						
HRQoL	EQ-5D/FACT-E/ GSE/PMS	X	X	X			X	X
HRQoL	Semi-structured interview			X			X	
Surgical Fear As measured by SFQ Time points Baseline post NCT Pre Surgery								
**Exploratory Endpoints**								
Clinical Health- Nutrition	GPS	X	X	X	X	X	X	
	Sarcopenia score	X	X	X			X	
	Foodbook 24	X	X	X			X	X
Morbidity	POMS				X	X		
	CD Classification						X	
Inflammatory Markers	WCC, CRP	X	X	X	X	X		

Abbreviations: *NCT* neoadjuvant chemotherapy, *6 MWT* 6-min walk test, *BMI* body mass index, *LOT-R* Life Orientation Test-Revised, HR *QoL Health realted* quality of life, *FACT-E* Functional Assessment of Cancer Therapy-Esophageal, *GSE * General Self-Efficacy, *PMS* Pearlin Mastery Scale, *SFQ* Surgical fear questionnaire, *GPS* Glasgow Prognostic Score, *POMS* Post-Operative Morbidity Score, *CD* Clavien-Dindo Classification, *WCC* white cell count, *CRP* C-Reactive Protein

### Primary outcome

#### Cardiorespiratory fitness

The primary outcome is measurement of cardiorespiratory fitness using the 6MWT assessed at the pre-surgery timepoint. The 6MWT is performed with participants walking up and down a 20 m course marked by cones for 6 min under instruction to cover as much ground as possible. The number of laps completed is recorded. A standard set of instructions is used as per the European Respiratory Society guidelines. The 6MWT is a validated assessment of cardiorespiratory function in clinical populations [30, 31]. A systematic review in 2016 demonstrated that field tests may be able to predict post-operative outcome; however, further validation work is merited [32].

### Secondary outcomes

#### Physical health

##### Strength

i) The sit to stand test. Participants sit on a chair (height 43–45 cm) with arms crossed across their chest, feet flat on the floor, parallel to each other, and approximately one shoulder width apart. Participants then stand up and sit down 10 times as quickly as possible and must fully extend their legs on each stand. The time taken to perform 10 repetitions is timed. Participants perform two trials, and the best trial is recorded [33].
ii) The handgrip test. This is measured using a hand dynamometer (Takei 5401 Hand Grip Dynamometer (digital)). The test is conducted in a standing position with the upper (dominant) arm tight against the participant’s trunk and the forearm at a right angle to the upper arm. The gripping handle is set to a comfortable width to ensure the participant can rest the fat pads of the phalanx of the four fingers on the handle. The participant is instructed to squeeze the handle with maximum force for 3 sec. The participant are asked to complete three trials with sufficient rest between each effort and an average is recorded [34].

##### Activity behaviour

Activity behaviour is assessed using a 7-day ActivPAL3 triaxial accelerometer. Participants in both groups are instructed to wear this device on the midpoint of the anterior aspect of the right thigh continuously for 7 days. The accelerometers do not provide participants with any feedback: data can only be analysed centrally by the lead researchers. Total activity counts per day as well as time in sedentary behaviour are recorded for both groups.

##### Body composition

Body mass index (BMI) is calculated in the standard manner.

##### Frailty

Frailty is assessed by the Risk Analysis Index. It has been reported as a valid tool for measuring frailty in surgical populations [35]. It provides a prospective pre-operative assessment of frailty in clinical practice and provides a score between 0 and 81, taking into account demographic, clinical and independence information.

##### Psychological health

This is assessed using a number of validated questionnaires and semi-structured interviews.

i. The Life Orientation Test-Revised (LOT-R) questionnaire assesses optimism and consists of ten items assessing expectancy of positive versus negative outcomes. Higher scores represent higher levels of optimism [36].
ii. The EQ-5D-5 L health questionnaire is a standardised measure of health status developed by the EuroQoL Group to provide a simple, generic measure of health for clinical and economic appraisal [37].
iii. Functional Assessment of Cancer Therapy-Esophageal (FACT-E) questionnaire is a health-related quality of life instrument validated in patients with oesophageal cancer. It is composed of a general component (FACT-G) and an Esophageal Cancer Subscale (ECS) [38].
iv. The Surgical Fear Questionnaire (SFQ) assesses participants fear of surgery and is a validated and reliable eight-item index of surgical fear consisting of two subscales: fear of the short-term consequences of surgery and fear of the long-term consequences of surgery [39].
v. The General Self Efficacy (GSE) and Pearlin Mastery Scale are highly reliable and validated measurements of self efficacy. GSE is a 10 item psychometric scale designed to assess optimistic self beliefs to cope with a variety of difficult demands in life and PMS consists of seven items designed to assess psychological coping resources (Mastery) [40].
vi. Semi-structured interviews explore patients’ perceptions of the surgical pathway.

**Exploratory outcomes**

*Nutritional Status* is assessed using the following tools:

i. The Glasgow Prognostic Score provides cancer prognosis based on serum biomarkers CRP and albumin [41].
ii. Foodbook-24, a web-based dietary tool consisting of a 24-h dietary recall and food frequency questionnaire [42].
iii. Standard care for all patients is to undergo a staging CT scan at the time of diagnosis and then a restaging CT scan after NCT. Sarcopenia is measured at these two time points using SliceOmatic software (Tomovision, Magog, Canada). At the L3 level, total skeletal muscle, subcutaneous fat and visceral fat will be measured. Skeletal muscle mass will be calculated as skeletal muscle / height (m)^2^ and will be recorded by two individuals, both of whom will be external to the trial group.

##### Post-operative morbidity outcomes

i. Post-operative Morbidity Score (POMS) is an 18-item tool that addresses morbidity relevant to the post-surgical patient [43].
ii. The Clavien-Dindo classification of surgical complications consists of seven grades that rank post-operative complication severity [44].
iii. The Comprehensive Complication Index [45] integrates all post-operative complications with their respective severities on a scale ranging from 0 (no burden from complications) to 100 (death).
iv. Patients undergoing oesophagectomy will have post-operative morbidity recorded as per the Esophagectomy Complications Consensus Group [28], with mortality being assessed at 30 days and 90 days.

##### Blood markers of inflammation

C- reactive protein and white cell count will be measured.

##### Health economic outcomes

An exploratory analysis will be made of the cost of the exercise intervention, and the net monetary benefit on health care costs and health care interactions that arise during the study will be calculated.

##### NCT toxicity

Rates of NCT toxicity, tolerance and compliance will be collected.

##### Patient and public involvement (PPI)

Patient and public volunteers are members of the trial steering committee, and their experience and input were used to help shape the study design. Volunteers attend quarterly trial steering meetings and receive monthly newsletters and trial management meeting minutes. They will assist with trial delivery and conduct as well as trial reporting. Additionally, they will guide our dissemination plan using social media, presentation at conferences, dissemination to patient advocacy groups and journal articles.

##### Safety

Adverse events are recorded in the relevant case report form by the lead site researcher. Fatal or life-threatening serious adverse events are reported within 24 h of the research team becoming aware of the event. The serious adverse events form documents the nature of the event, date of onset, severity, corrective therapies given, outcome and causality (i.e., unrelated, unlikely, possibly, probably or definitely). Any queries relating to adverse event reporting will be directed to the principal investigator.

#### Data analysis

##### Sample size calculation

The sample size calculation was based on results from a recent publication by Minnella et al. [46] who identified a pre-operative increase in 6MWT of 60 m from a baseline score of 450 m, which is an improvement of approximately 13%. Assuming a similar baseline score, a 15% difference can be detected with a *p* value of 0.05 and power of 80% with a sample of 26 participants with full data in the two groups. With an anticipated 20% drop out, recruitment of 62 participants is anticipated.

##### Statistical analysis

The analysis will be performed as an intention-to-treat analysis. No interim analysis will be conducted. Data validity will be conducted prior to analysis and corrected as appropriate.

The study population will be described separately for the two randomised groups using variables obtained at baseline. The variables will be described as mean (SD) and numbers (%) as appropriate.

The primary analysis of the primary outcome will be conducted using t-tests of independent group mean differences in 6MWT. The mean difference and 95% confidence interval will be reported and illustrated graphically. Individual change in 6MWT will be calculated from baseline and compared at different time points using t-tests.

The secondary analysis of the primary outcome will use mixed-level analysis with intervention group, time point and interaction of intervention and time points. This analysis will include baseline score for the outcome measure as a covariate. The estimated parameter for the interaction variables will be interpreted as the difference-in-difference between the two groups over time. A separate analysis will explore potential differences in the intervention group between participants who received the intervention at a training centre and those who trained at home. This analysis will be expanded to include descriptive baseline variables such as sex and age. The secondary analysis will use mixed-level analysis and include baseline score and baseline characteristics as covariates.

The cost-effectiveness analysis will be conducted from a societal perspective over the duration of the trial period. No extrapolation of long-term economic outcomes is planned. The EQ-5D-5 L data reported at each time point will be used to estimate quality-adjusted life years using time-weighted utility scores. The utility scores will be calculated for each individual at each data point using the Irish scoring algorithm for EQ-5D-5 L [47]. The area under the curve denotes the QALY, and incremental QALY is determined as the mean group difference.

The cost of the intervention and subsequent health care resource use will be calculated for each individual using average cost per participant for the intervention programme and self-reported data on healthcare utilisation. Unit costs will be obtained from national sources and assigned to the resource utilisation and aggregated over the whole trial period for each individual. Net monetary benefit (NMB) will be estimated as the cost minus the QALY gain multiplied by an assumed threshold value per QALY.

##### Missing data

Participants with missing data either because of early drop-out, loss to follow-up or missed participation in the data collection can bias the results. By design, no data will be missing at baseline because only participants with complete baseline data will be randomised.

Missing variables in outcome measures will be handled according to instrument developers’ guidelines. As a general rule, if more than 20% of the items of an instrument are missing, the summary score will be assigned as missing. Missing data will be reported as part of the summary presentation of the raw data. Logistic regression will be used to explore whether participants with missing data have different characteristics than the completers or whether missing data can be assumed missing by random.

##### Procedures for data checking and entering

Data will be double data entered, and data validation will take place according to the procedures set out in the data management plan and data validation plan. Prior to any statistical analysis, all variables will be checked for the number of missing values, impossible values and improbable values. Impossible and improbable values will be defined by clinical opinion. Improbable values will also include values that are outside three standard deviations of the mean value. Any questions regarding the data will go back to the data manager. Descriptive statistics will be calculated for all variables, and distributional assumptions will be checked.

The Standard Protocol Items-Recommendations for Interventional Trials (SPIRIT) table provides an overview of the study conduct, review, reporting and interpretation and is presented in Table 2. The final report will follow the Consolidated Standards of Reporting Trials (CONSORT), as well as the Template for Intervention Description and Replication (TIDieR).

**Table 2:** The Standard Protocol Items-Recommendations for Interventional Trials (SPIRIT)

Section/item	Item No	Description	Addressed on page number
**Administrative information**			
Title	1	The effect of a pre- and post-operative exercise programme versus standard care on physical fitness of patients with oesophageal and gastric cancer undergoing neoadjuvant treatment prior to surgery (The PERIOP-OG Trial): Study protocol for a randomised controlled trial.	1
Trial registration	2a	ClinicalTrials.gov Identifier: NCT03807518	4
	2b	N/A	
Protocol version	3	1 November 2019 Version 2	4
Funding	4	Beaumont Hospital Foundation Trust, Oesophageal Cancer Fund, Royal College of Surgeons in Ireland.	25
Roles and responsibilities	5a	WR, NMcC, LL conceived the study. WBR, NMcC, PAC, MA, TJM, CGC, OM, LL, RT, JB, PG, CT and JS contributed to the study design. WR, RT, LL and JB drafted the manuscript, which underwent revision by all other authors.	25
	5b	Royal College of Surgeons Ireland, 123 St Stephens Green, Dublin, Ireland Sponsor had no role in study design	1
	5c	Mr. William Robb, Principal Investigator Dr. Noel McCarrfey, Co-investigator Mr. Thomas Murphy, Co-investigator Mr. Chris Collins, Co-investigator Prof. Oliver McAnena, Co-investigator Mr. Paul A Carroll, Co-investigator Dr. Lisa Loughney, Coordinator and exercise lead Dr. Roisin Tully, Coordinator Mr. Jarlath Bolger, Recruiting and consenting patients Mr. Mayilone Arumugasamy Co-investigator Prof. Pamela Gallagher, Psychological lead Dr. Claire Timon, Nutritional lead Prof. Niall Moyna, Exercise specialist Prof. Jan Sorensen, Health Economist Prof. Arnold Hill, Professor of Surgery	1
	5d	Steering committee: William Robb, Lisa Loughney, Roisin Tully Data management team: Lisa Loughney, Roisin Tully, Jan Sorenson, Jarlath Bolger	
**Introduction**			
Background and rationale	6a	Oesophagogastric cancers are a considerable health burden. In the past 10 years the 5-year survival for both cancers has doubled. This is due to a number of factors including advances in neoadjuvant and adjuvant chemotherapy and radiotherapy. However, physical fitness significantly declines as a result of neoadjuvant and adjuvant therapy. From studies in other cancers, perioperative training is known to improve physical fitness, yet little research has been performed on its effects in those with upper oesophagogastric cancers. Therefore, the aim of the PERIOP-OG trial is to investigate the effects of a community-based exercise training programme (delivered in a leisure centre or at home, depending on the patient location) pre- and post–operatively compared to usual care on cardiorespiratory fitness and other physical, psychological and clinical health outcomes in people with confirmed oesophagogastric cancer.	5
	6b	The usual-care control group (usual care – no formal exercise training) receive routine care throughout their cancer pathway from diagnosis to surgical resection. No specific advice about exercise training is offered.	9
Objectives	7	The aims of this study were to evaluate the following hypotheses: Primary hypothesis: A structured community-based exercise programme compared with a usual care control group (usual care – no formal exercise training) will result in a clinically significant increase in cardiorespiratory fitness assessed using a 6-min walk test between the baseline and the pre-surgery time point. Secondary hypotheses: A structured community-based exercise programme compared with a usual care control group (usual care – no formal exercise training) will result in improvements in other physical health outcomes assessed using upper and lower body strength test, activity behavior monitoring, body mass index, frailty, and psychological health questionnaires assessed at 5 time points baseline, post NCT, pre-surgery, 4 weeks and 10 weeks after surgery as well as semi-structured interviews assessed pre and post-surgery. Exploratory Endpoints: Post-operative morbidity, comprehensive complication index, hospital length of stay, nutritional status, immune and inflammatory markers, cancer staging, response to NCT and a medico-economics analysis of cost effectiveness of the exercise intervention on reducing health care costs and burden additionally rates of NCT toxicity, tolerance and complicance will be measured.	7
Trial design	8	Parallel group randomised 1:1 controlled multi-centre trial.	9
**Methods: Participants, interventions, and outcomes**			
Study setting	9	Three Irish Health Services Executive (HSE) hospitals are recruiting to this trial: Beaumont Hospital Dublin, Mercy University Hospital Cork (MUHC) and University Hospital Galway (UHG). Assessments and exercise training are being delivered at several sites. For Beaumont hospital Dublin: ExWell Medical; for MUHC, to cater for a large area: Cork Leisure World, Waterford Institute of Technology, Heartwise for Health and the University of Limerick; and for UHG, Cancer Care West gym.	7
Eligibility criteria	10	Eligibility criteria for inclusion at cancer diagnosis include the following: age ≥ 18 years, with multidisciplinary team (MDT) referral for neoadjuvant chemotherapy or neoadjuvant chemoradiotherapy (NCT) prior to planned oesophagectomy or gastrectomy; with confirmed MDT evidence of adenocarcinoma or squamous cell cancer of the oesophagus, oesophago-gastric junction or stomach requiring planned surgical resection; with recorded measurement (endoscopic or otherwise) that the tumour starts more than 5 cm below crico-pharyngeus. Exclusion criteria include the following: inability to give informed consent; inability to participate in exercise training (unable to perform 6MWT); patients with high-grade dysplasia (squamous cell or adenocarcinoma), distant metastatic disease at time of enrolment or during their NCT therapy, or showing evidence of previous/concomitant malignancy that would interfere with this treatment protocol; or pregnancy.	8
Interventions	11a	Participants are randomised (1:1) to either a structured exercise training programme or usual care control group at baseline	9
	11b	The intervention will be discontinued for a given participant should they no longer wish to participate.	10
	11c	Strategies to improve adherence to intervention protocols include the following: the participants will meet the instructors face to face at the structured exercise sessions, and in addition, they will receive phone calls from the team to monitor their progress and answer any questions they may have on a regular basis.	10
	11d	All routine cancer care is permitted for both groups	9
Outcomes	12	The primary endpoint is cardiorespiratory fitness measured by the 6-min walk test between the baseline assessment and the pre-surgery time point. Secondary endpoints include the measurement of cardiorespiratory fitness, physical health assessed using upper and lower body strength tests, activity behaviours, body mass index, and frailty; and psychological health assessed using health-related quality of life questionnaires and semi structured interviews. Exploratory endpoints include post-operative morbidity, comprenhensive complication index, hospital length of stay, nutritional status, immune and inflammatory markers, cancer staging, response to NCT, medico-economics analysis of cost effectiveness of the exercise intervention on reducing health care costs and burden additionally rates of NCT toxicity, tolerance and compliance.	13
Participant timeline	13	Outcome measurements are taken for all participants at baseline, post-NCT, pre-surgery, 3 days post-surgery, 5 days post-surgery, 4 weeks post-surgery and 10 weeks post-surgery.	7
Sample size	14	The sample size calculation was based on results from the Minnella et al. study, which identified a pre-operative score gain in 6 MWT of 60 m from a baseline score of 450 m (standard deviation 85 m), which is an approximate 13% improvement. Assuming a similar baseline score, a 15% score gain can be detected with *p* value of 0.05 and power 80% with a sample of 26 participants with full data in two groups. With an anticipated 20% drop-out, recruitment of 62 participants is anticipated.	18
Recruitment	15	Inclusion of three clinical sites and seven exercise sites with the additional option of a home programme.	7
**Methods: Assignment of interventions (for controlled trials)**			
Allocation			
sequence generation	16a	Randomisation is performed using a central data management to generate a random allocation sequence (1:1).	9
Allocation concealment mechanism	16b	Randomization allocation is stored in opaque envelops at the lead exercise site (ExWell Medical, Dublin).	9
Implementation	16c	Randomisation is generated by the lead coordinator and exercise lead (LL).	9
Blinding (masking)	17a	Due to the nature of the intervention, blinding of patients or psysiological assessors is not possible. Treating surgeons and their teams are blinded to randomisation as is the primary analyst.	9
	17b	Unblinding will not be permissible	9
**Methods: data collection, management, and analysis**			
Data collection methods	18a	The outcomes are listed in Table 1: Outcomes and assessment measures.	13
	18b	The participants are contacted by telephone during the intervention to promote participant retention and complete follow-up. Baseline data to be collected for participants who discontinue or deviate from intervention protocols, unless they withdraw consent.	13
Data management	19	Data will be double data entered, and data validation will take place according to the procedures set out in the data management plan and data validation plan. Prior to any statistical analysis, all variables will be checked for the number of missing values, impossible values and improbable values. Impossible and improbable values will be defined by clinical opinion. Improbable values will also include values that are outside three standard deviations of the mean value. Any questions regarding the data will go back to the data manager. Descriptive statistics will be calculated for all variables, and distributional assumptions will be checked.	20
Statistical methods	20a	The analysis will be performed as an intention-to-treat analysis. No interim analysis will be conducted. Data validity will be conducted prior to analysis and corrected as appropriate. This includes tabulation of discrete score values and graphical representation of continuous variables (e.g., histograms and box plots). The study population will be described separately for two randomised groups using variables obtained at baseline. The variables will be described as mean (SD) and numbers (%) as appropriate. The primary analysis of the primary outcome will be conducted as t-tests of independent group mean differences in 6MWT at each time point. The mean difference and 95% confidence interval will be reported and illustrated graphically. Individual change in 6MWT will be calculated from baseline and compared at different time points using t-tests. In addition, binary outcome variables indicating ability to walk more than the combined median distance at baseline will be constructed, and the group distribution will be tested at different time points using chi-squared tests.	18
	20b	The secondary analysis of the primary outcome will use mixed-level analysis with intervention group, time point and interaction of intervention and time points. This analysis will include baseline score for the outcome measure as covariate. The estimated parameter for the interaction variables will be interpreted as the difference-in-difference between the two groups over time. A separate analysis will explore potential differences in the intervention group between participants who received the intervention at a training centre and those who trained at home. This analysis will be expanded to include descriptive baseline variables such as sex and age. The secondary analysis will use mixed-level analysis and include baseline score and baseline characteristics as covariates. The cost-effectiveness analysis will be conducted from a societal perspective over the duration of the trial period. No extrapolation of long-term economic outcomes is planned. The EQ-5D-5 L data reported at each time point will be used to estimate quality-adjusted life years using time-weighted utility scores. The utility scores will be calculated for each individual at each data point using the Irish scoring algorithm for EQ-5D-5. The area under the curve denotes the QALY, and incremental QALY is determined as the mean group difference. Cost of the intervention and subsequent resource use will be calculated for each individual using average cost per participant for the intervention programme and self-reported data on healthcare utilisation. Unit costs will be obtained from national sources and assigned to the resource utilisation and aggregated over the whole trial period for each individual. Net monetary benefit (NMB) will be estimated as the cost minus the QALY gain multiplied by an assumed threshold value per QALY. The NMB estimates will also be analysed using regression methods to account for variation in group characteristics and to identify sub-populations where the intervention might have an incremental cost-effectiveness ratio.	
	20c	Participants with missing data, because of early drop-out, loss to follow-up or missed participation in the data collection, can bias the results. By design, no data will be missing at baseline because only participants with complete baseline data will be randomised. Missing variables in outcome measures will be handled according to instrument developers’ guidelines. As a general rule, if more than 20% of the items of an instrument are missing, the summary score will be assigned as missing. Missing data will be reported as part of the summary presentation of the raw data. Logistic regression will be used to explore whether participants with missing data have different characteristics than the completers or whether missing data can be assumed missing by random. If a pattern in missing data can be observed, missing data will be handled using “multiple imputation” techniques where missing variables are predicted in multiple dataset using descriptive variables identified as important covariates for missing data (sex, age, intervention group and baseline score).	19
**Methods: monitoring**			
Data monitoring	21a	Data are monitored after the first complete patient at each site to ensure high-quality data. Data will be double data entered, and data validation will take place according to the procedures set out in the data management plan and data validation plan. Prior to any statistical analysis, all variables will be checked for the number of missing values, impossible values and improbable values. Impossible and improbable values will be defined by clinical opinion. Improbable values will also include values that are outside three standard deviations of the mean value. Any questions regarding the data will go back to the data manager. Descriptive statistics will be calculated for all variables, and distributional assumptions will be checked.	20
	21b	No interim analysis will be conducted.	18
Harms	22	Adverse events will be recorded in the relevant case report form by the researcher. Fatal or life-threatening serious adverse events are reported within 24 h of the research team becoming aware of the event. The serious adverse events form documents the nature of the event, date of onset, severity, corrective therapies given, outcome and causality (i.e., unrelated, unlikely, possibly, probably, or definitely). Queries relating to adverse event reporting will be directed to the chief investigator in the first instance.	17
Auditing	23	Complete compliance will occur with any auditing processes required by sponsor.	17
**Ethics and dissemination**			
Research ethics approval	24	Beaumont Hospital Ethics (Medical Research) Committee REC Ref: 18/58 Dublin City University Research Ethics Committee Ref: DCUREC/2018/255 University Hospital Galway Clinical Research Ethics Committee Ref: C. A 2160 Mercy Hospital Cork CREC Review Reference Number: ECM 4 (mm) 19/04/19 Waterford Institute of Technology REF:WIT2019REC0011	24
Protocol amendments	25	All site leads will be contacted by telephone if a significant amendment is made to the protocol. The amended protocol will then be emailed to all site leads.	
Consent or assent	26a	The study will be discussed with patients after their initial diagnosis is known but before neo-adjuvant treatment has started. No patient will have the study discussed with them on the day that they find out their diagnosis. Potentially eligible patients will have the study discussed with them by the principal investigator, or a nominated senior non-consultant hospital doctor at their next OPD appointment. Interested patients will receive an information leaflet and consent form for the study. After a period of 72h, patients will be contacted by telephone to confirm their interest in the study. Consent forms will be signed at their patient visit.	25
	26b	Additional explicit consent will be sought for collection and use of participant data and biological specimens in ancillary studies.	25
Confidentiality	27	Data will be entered with all direct patient identifiers removed; patients will be identified by study codes. All physiological data are held in an encrypted format. All data will be stored on a secure password protected desktop in a secured locked room.	20
Declaration of interests	28	The authors declare that they have no competing interests	25
Access to data	29	The data will only be accessed by the designated members of the research team.	
Ancillary and post-trial care	30	After the trial, the structured exercise classes will continue, and participants may continue to use the facilities.	
Dissemination policy	31a	The results will be published in peer-reviewed journals and disseminated at conferences and scientific meeting internationally. The findings of the trial will also be published in patient information magazines and booklets as informed with the help of the patient public involvement group.	
	31b	Authorship eligibility guidelines will be discussed and agreed with all contributors prior to publication, and the use of professional writers is not intended.	
	31c	No plans exist to grant public access to the full protocol, participant-level dataset, and statistical code.	
**Appendices**			
Informed consent materials	32	Model consent form and other related documentation are given to the participants and authorised surrogates.	
Biological specimens	33	Plans for collection, laboratory evaluation, and storage of biological specimens for genetic or molecular analysis in the current trial and for future use in ancillary studies, if applicable. (Not applicable)	

## Discussion

Exercise programmes can impact the outcomes of patients undergoing neoadjuvant therapy and surgery for oesphagogastric cancers in a variety of ways. They appear to improve patient functional status, HRQoL and, possibly, peri-operative morbidity [20]. Functional capacity may significantly improve during NCT when a prehabilitation programme is instituted [46, 48–54]. The use of exercise interventions and their timelines are inconsistently reported in the published literature as are the associated outcome measures that have included maximal inspiratory pressure [48–50], 6MWT [46, 52], gait speed [53], FEV1/FVC [51], VO2max and hand grip strength [49, 50]. The heterogeneity in the reported outcomes clearly makes it difficult to compare outcomes between studies. The PERIOP-OG trial aims to address this heterogeneity by including a number of outcome measures to examine cardiorespiratory fitness as well as physical strength. This will provide simple, easily reproducible measures of physical fitness that require minimal specialist equipment and can be widely reproduced in future comparable studies.

Within this trial, patient outcomes are comprehensively recorded during NCT, hospitalisation for resectional surgery and during post-operative recovery. Although the trial is not powered to investigate morbidity as a primary outcome, it may yield some insights as to the effect of the prescribed PERIOP-OG exercise programme on patient morbidity before, during and after surgery.

Quality of life and psychological outcomes are central to improving patient tolerance of NCT and oesophagogastric resection. Both also are recognised to be of increasing importance in survivorship. To date, there has been limited focus on whether exercise prescription can impact these outcomes. Most of the available data in this area are derived from post-operative rehabilitation interventions, which includes patients who may be a number of years from their surgery [19, 55, 56]. The PERIOP-OG trial includes multiple validated measures of patient outcomes, including the LOT-R, EQ-5D-5, FACT-E, GSE, PMS, SFQ, in addition to semi-structured interviews exploring patients’ experience of their peri-operative and surgical pathway. This will provide a comprehensive overview of the influence of this exercise intervention on psychosocial outcomes both before surgery and then after surgery as patients enter recovery and survivorship.

Importantly, any exercise interventions must not impact nutritional status. Specialised multidisciplinary input is available in all participating centres, with dedicated dieticians being involved in each patient’s care and with frequent measures being taken of BMI and nutritional status.

Ideally, all patients would attend supervised training exercise sessions. This is not feasible given the multi-centre nature of this trial, and the geographical distribution of patients. In an effort to maximise gym utilisation and the CBEP, providers will be trained in delivering the PERIOP-OG programme at multiple sites nationwide. This will reduce patients’ commute times and will improve compliance with interventions. For those unable to attend routinely for training, a HBEP is provided. This is a pragmatic approach to the delivery of an exercise programme for all patients, including those who live rurally or remotely from exercise centres. The use of patient activity trackers, frequent patient contact, exercise logbooks and motivational reviews will aim to maximise patient compliance. Although heterogeneity exists including home-based and centre-based exercise programmes, this could be a sustainable model for prehabilitation of surgical-oncology patients in the future.

PERIOP-OG provides a comprehensive programme to examine a strategy of peri-operative exercise in oesophagogastric cancer patients undergoing neoadjuvant treatment followed by curative surgery. This trial will be the first to examine the outcomes of peri-operative exercise training in this patient cohort. The interventions studied are easily reproducible and may provide a standardised framework for the prescription of exercise in oesophagogastric cancer patients.

## Trial status

The trial registration number is ClinicalTrials.govNCT03807518. Protocol Version 2 31 Oct 2019. The PERIOP-OG trial began recruitment on 1 March 2019. The anticipated end date is May 2020. To date 29 participants have been recruited.

## Data Availability

The datasets used and/or analysed during the current study will be available from the corresponding author on reasonable request.

## Abbreviations

NCT: Neoadjuvant chemotherapy
MDT: Multi-disciplinary team
6 MWT: 6-minute walk test
HRQoL: Health-related quality of life
LOT-R: Life Orientation Test-Revised
FACT-E: Functional Assessment of Cancer Therapy-Esophageal
GSE: General self-efficacy
PMS: Pearlin Mastery Scale
BMI: Body mass index
POMS: Post-Operative Morbidity Score
CD: Clavien-Dindo Classification
WCC: White cell count
CRP: C-reactive protein
QALY: Quality-adjusted life year
NMB: Net monetary benefit
SPIRIT: Standard Protocol Items-Recommendations for Interventional Trials
CBEP: Centre-based exercise programme
HBEP: Home-based exercise programme
REP: Rate of perceived exertion
